# The determinants of public acceptance of telemedicine apps: an innovation diffusion perspective

**DOI:** 10.3389/fpubh.2023.1325031

**Published:** 2023-12-13

**Authors:** Dong Liu, Sangbum Son, Junwei Cao

**Affiliations:** ^1^Department of Global Business, Yeungnam University, Gyeongsan, Republic of Korea; ^2^Department of Business, Yangzhou University, Yangzhou, China

**Keywords:** telemedicine apps, public acceptance, diffusion of innovation theory, theory of perceived value, structural equation modeling

## Abstract

With the rapid advancement of information technology, telemedicine apps have gradually become an indispensable tool for providing patients with more convenient, efficient, and accessible healthcare services. However, the successful implementation of these apps largely depends on widespread acceptance among the public. To thoroughly investigate the factors influencing the public’s acceptance of these apps and the relationships between these factors, this study developed a theoretical model based on the Diffusion of Innovation theory and the Theory of Perceived Value. To validate this model, we conducted a survey of 387 residents in Beijing, China, and employed structural equation modeling to analyze the collected data. The research findings indicate that attributes of innovation diffusion, including relative advantage, compatibility, complexity, trialability, and observability, significantly and positively influence the public’s perceived value. Particularly noteworthy is that perceived value partially mediates the relationship between innovation attributes and public acceptance, emphasizing the crucial role of perceived value in the public decision-making process. This study employed a theory-driven approach to elucidate the acceptance of telemedicine apps and offers fresh insights into the existing literature. By integrating the research paradigms of innovation diffusion and customer perceived value, we provide a coherent explanation of how individual cognitive processes lead to acceptance behavior. In summary, this research enriches the existing theoretical studies on the acceptance of telemedicine apps and holds positive implications for healthcare practice.

## 1 Introduction

In the current context of the information society, the field of healthcare is undergoing a wave of digital transformation (1–3). With the rapid development of information and communication technology, particularly the emergence of next-generation communication technologies such as 5G/6G, it is anticipated that by 2030, the global telemedicine market will grow to $286.22 billion [Fortune Business (4)]. Telehealth refers to the provision of medical services at locations distant from the patient, encompassing three key concepts: telecare, telemetry, and telemedicine (5). Telecare enables individuals to receive nursing services remotely in the comfort of their homes. Telemetry utilizes sensors, remote processing systems, and wireless networks to achieve real-time monitoring of patients’ health conditions. Lastly, telemedicine successfully overcomes geographical barriers between doctors and patients by facilitating remote consultations (teleconsultation). This study will primarily focus on the concept of telemedicine in its entirety.

Against the backdrop of this digital wave, Telemedicine Apps (TA)are increasingly gaining prominence as a significant innovation in the healthcare sector (6, 7). TA is a category of healthcare software applications that utilize digital communication technology to provide remote medical services to both patients and healthcare professionals (6, 8). These apps offer several significant advantages and can have a positive impact on society. Firstly, TA leverage mobile devices and internet connectivity to deliver convenient healthcare services and health management, addressing the imbalance of medical resources (7, 9). Secondly, these apps enable patients to access medical consultations and treatment anytime and anywhere, greatly enhancing the accessibility of healthcare services, especially for those living in remote areas or with limited mobility (10, 11). Particularly during the COVID-19 pandemic, the importance of TA became even more pronounced. In situations where the pandemic led to shortages of medical resources, TA provided a safe and efficient means of healthcare delivery, supporting public health measures, reducing the need for patients to physically visit hospitals, and effectively lowering the risk of infection (12–14).

However, like any innovation, the widespread adoption and application of TA also face a variety of issues and challenges. These issues and challenges may impact the extent to which the public embraces them, including but not limited to concerns regarding data security and privacy (15–17), technological barriers stemming from the digital divide (16, 18, 19), medical liability and legal issues (16, 20, 21), as well as concerns about service quality and accuracy (7, 22).

In recent years, the academic community has embarked on extensive research aimed at exploring various factors influencing the acceptance of TA by the public. These studies cover several key areas, including the development of new regulations to address ethical issues related to health applications (22), as well as the design of effective communication channels to enhance the user experience of TA, particularly focusing on patients in remote areas (23). During the global pandemic, some research has also focused on investigating customer emotional responses to TA during the COVID-19 crisis, providing valuable insights into public attitudes (24). Additionally, there have been studies evaluating the usability of user interface designs for TA deployed during the COVID-19 pandemic to ensure their maximum effectiveness in emergency situations (25). Furthermore, attention has been given to the barriers and facilitators faced by the 53 member countries of the World Health Organization (WHO) European Region in using TA (17).

However, despite the deepening research on TA, there has been limited focus on using theories to explain the public’s acceptance of TA. The application of theories can provide a coherent and logical framework for understanding people’s attitudes and behaviors toward the acceptance of TA. For example, a recent study explored the application of the Technology Acceptance Model (TAM) in TA during the COVID-19 pandemic, emphasizing the impact of external factors such as privacy concerns and trust on the relationship between perceived usefulness and perceived ease of use (26). Another study, in the context of emerging markets, utilized the Innovation Resistance Theory to investigate barriers to adoption and intentions to continue using TA (7). Furthermore, there is currently insufficient research in the academic community that focuses on exploring the perceived value of TA among the public. By examining the actual or perceived value experiences that TA provide in people’s lives, we can better understand the acceptance behavior of the public toward this innovative technology.

To address the gaps in existing research, this study seeks to elucidate the public’s acceptance of TA through the lenses of the Innovation Diffusion Theory and the Perceived Value Theory. The Innovation Diffusion Theory underscores that individuals’ reception of novel products or services is shaped by five pivotal factors: relative advantage, compatibility, complexity, trialability, and observability (27, 28). This theoretical framework is highly pertinent to our investigation since TA can be viewed as an innovative facet of healthcare services (6, 7).

Conversely, the Perceived Value Theory posits that if TA can offer the utmost utility to users, it will augment the public’s willingness to adopt the application (29). Research indicates that the perceived value of a product or service predominantly hinges on users’ assessments of its attributes (30–32). Thus, to comprehensively probe the impetuses and underlying mechanisms behind the public’s acceptance of TA, this study will delve into the following pivotal research inquiries:

RQ1. How do the five elements of innovation diffusion impact the perceived value of TA among the public?

RQ2. What is the influence of perceived value on the public’s acceptance of TA? Additionally, what mechanisms mediate the relationship between the five elements of innovation diffusion and the public’s acceptance of TA?

The organization of this paper is as follows. The first section introduces the theoretical background of TA and the Diffusion of Innovation Theory, along with the Perceived Value Theory. The second section presents the model and hypotheses based on the theory and research objectives. The third section conducts empirical analysis, including surveys and investigations, outlining the methodology and hypothesis validation. The fourth section comprises the discussion and results, encompassing key findings, theoretical and practical contributions, as well as limitations and suggestions for future research.

## 2 Literature review and theoretical background

### 2.1 Telemedicine apps

TA are healthcare software applications that utilize Information and Communication Technology (ICT) to connect healthcare professionals with patients, aiming to provide healthcare services remotely (22, 23). These applications typically offer various functionalities such as video calls, messaging, image sharing, and medical record storage to facilitate effective communication and collaboration between doctors and patients. By connecting patients and healthcare professionals via the internet, TA enable them to address health-related issues, including prevention, diagnosis, treatment, and follow-up, overcoming logistical and geographical barriers (33).

TA have made significant strides in multiple healthcare domains, including general medicine, mental health, dermatology, and obstetrics and gynecology (34, 35). These applications offer patients a more convenient healthcare option, particularly for those who have difficulty accessing hospitals or clinics, which is of paramount importance (36). Furthermore, they enhance the efficient utilization of healthcare resources, alleviate the burden on healthcare systems, and foster close collaboration between patients and healthcare teams (6). In special circumstances, such as during the COVID-19 pandemic, the role of these applications is especially prominent as they provide pathways for delivering critical medical support and services (25, 37). Globally, the rise of TA significantly improves the convenience and accessibility of healthcare services, especially for patients facing limited healthcare resources or residing in remote areas (35, 38).

However, caution must be exercised when using TA to ensure healthcare quality and safety (7, 22). It is important to note that TA should not replace all healthcare services but should serve as a complement (39). Particularly in cases involving emergency medical situations or requiring physical examinations, seeking in-person medical treatment remains necessary.

Some well-known TA include Teladoc Health (United States), Doctor On Demand (United States), Amwell (United States), Ping An Good Doctor (China), Babylon Health (United Kingdom), KRY (Sweden), and others. These applications are typically available in different regions and countries, and their specific features and availability may vary by location. The ongoing development of these applications provides new tools and resources for the healthcare sector, with the potential to further drive improvements and innovations in medical services.

### 2.2 Diffusion of innovation theory

The Diffusion of Innovation (DOI) Theory is a significant theory in the field of social sciences, first proposed by American sociologist Everett Rogers in 1962. It continues to be widely cited and applied in research and practice across various domains (28, 40–41).

In the DOI Theory, innovation is defined as a concept, practice, or thing perceived as novel by individuals or other adopting units (28). The process of innovation diffusion encompasses various types of adopters, each exhibiting different attitudes and behaviors when confronted with innovation. Adopters are typically categorized into five groups: innovators, early adopters, early majority, late majority, and laggards, with each group playing a unique role in the spread of innovation. The dissemination channels play a pivotal role in the DOI and include various avenues such as media, social networks, and word-of-mouth communication. Different dissemination channels can significantly impact the speed and coverage of innovation diffusion. The process of innovation adoption typically goes through several consecutive stages, including awareness, interest, evaluation, trial, and adoption, with different adopters potentially adopting innovation at different stages. Furthermore, the characteristics of innovation also play a crucial role in the innovation diffusion process. These characteristics encompass relative advantage, compatibility, complexity, trialability, and observability.

In the context of this study, TA are considered an innovation in the field of healthcare services (6, 7). According to the DOI Theory, the dissemination and adoption of innovations depend on the characteristics of the innovation (28). These characteristics include relative advantage (the degree of advantage of TA over traditional healthcare methods), compatibility (the degree of alignment between TA and users’ medical practices, beliefs, and values), complexity (whether using TA is easy to understand and operate), trialability (whether TA can be tested on a small scale), and observability (whether the effects of TA are easily visible to others), among others. These characteristics will help us better understand the acceptance of TA in the healthcare field. By applying the framework of the DOI Theory, we can delve into the acceptance process of TA, identify key influencing factors, and provide robust support for decision-making in the healthcare domain.

### 2.3 Perceived value theory

The Perceived Value Theory (PVT) constructs a theoretical framework aimed at explaining how consumers assess and perceive the value of products or services. The core idea of this theory revolves around the notion that consumers engage in a comprehensive evaluation based on the perceived benefits of a product or service relative to their investment, which ultimately forms the basis for their purchase or usage decisions (29).

However, defining and measuring perceived value has always been a rather complex task. Nonetheless, the most common definition describes it as a ratio or balance between quality and price, commonly referred to as “value for money” (43). With further research, the concept of perceived value has evolved from its initial single-dimensional perspective into a multi-dimensional one (44). This multi-dimensional concept encompasses not only evaluations of the attributes of the product or service itself but also perceptions of external factors, while also integrating both rational and emotional perspectives (45). Among the various dimensions of perceived value concepts, utilitarian value, emotional value, and social value are widely recognized as primary dimensions (43, 46, 47).

The PVT has garnered widespread academic interest and has been applied across various disciplinary domains to elucidate consumer behavior in different contexts. In the current literature, researchers have employed PVT to assess various consumer decisions, such as the determination of consumers to continue using live streaming services (48), the sustained intent to adopt Virtual Reality (VR) technology (49), factors influencing willingness to pay extra for energy-efficient appliances (50), word-of-mouth behavior in WeChat settings (51), and revisitation and word-of-mouth intentions for organic food restaurants (52). These studies underscore that PVT has evolved into a robust theoretical framework for dissecting consumer behavior and intentions. Importantly, it is well-suited for examining the public’s acceptance of TA.

The PVT posits that products or services with higher perceived value are more likely to be accepted and continuously used by the public (43, 53). In the context of TA, perceived value becomes a crucial metric for assessing the public’s acceptance of these applications. Therefore, if the public perceives TA as offering value in terms of utilitarian, emotional, and social dimensions, they are more likely to accept and continue using the application.

## 3 Research model and hypothesis

### 3.1 Research model

**Figure 1:**
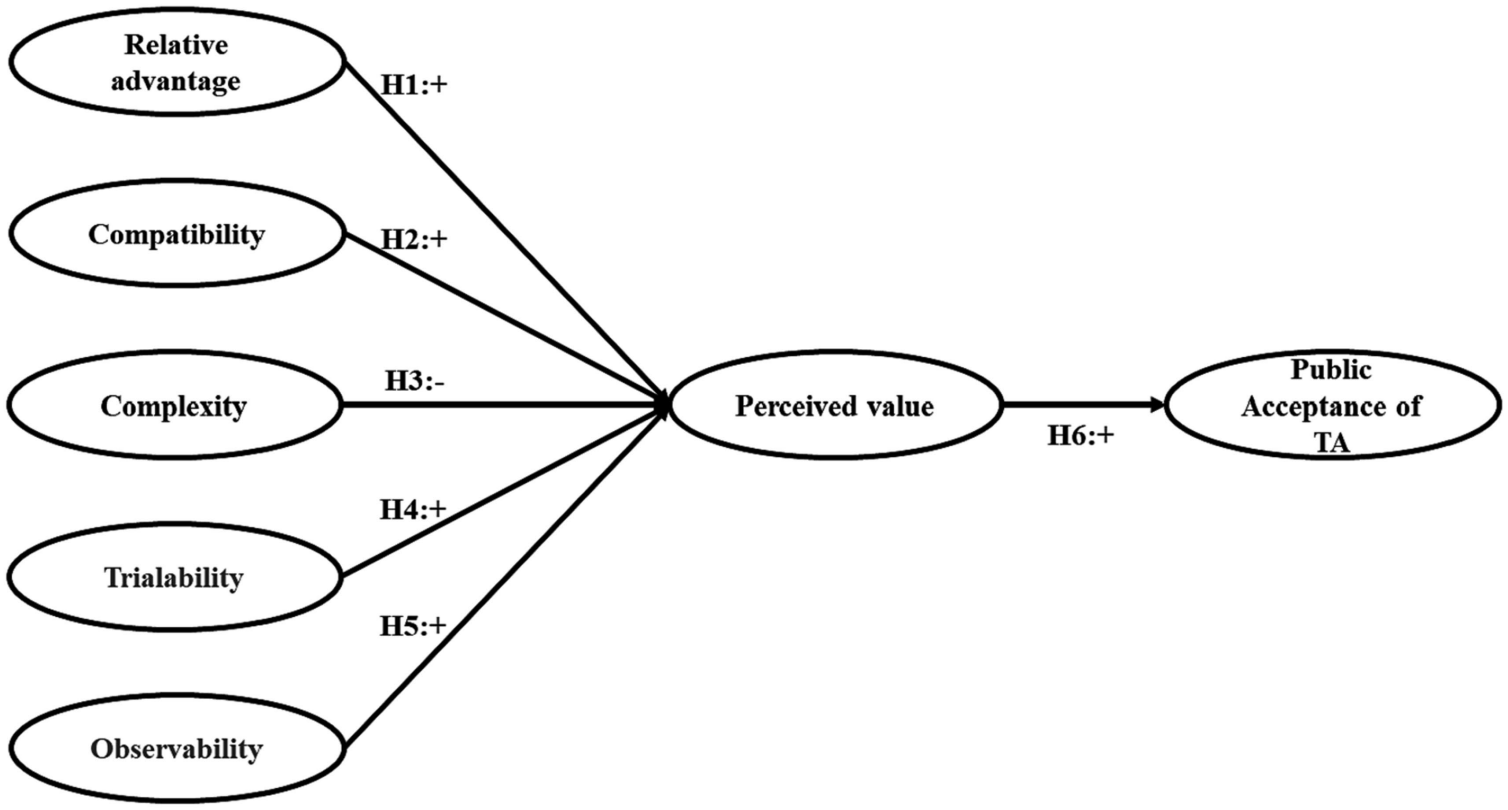
Research model.

This study aims to delve into the motivations and influencing mechanisms behind the public’s acceptance of TA. In this context, we have developed the following research model, as depicted in Figure 1, based on the foundations of the DOI Theory and the PVT, with appropriate adjustments made to align with the research objectives.

### 3.2 Hypothetical development

#### 3.2.1 Relative advantage

Relative advantage is one of the crucial factors in assessing the adoption of an innovation or technology (53, 54). Relative advantage refers to the extent to which an innovation is perceived as better than the idea it supersedes (28). This concept is akin to the notion of perceived usefulness in technology acceptance models (55–58). Numerous studies have demonstrated that the relative advantage of a product or service significantly influences consumers’ perceived value of the product or service (59–61).

In the context of this study, TA offer distinct advantages over traditional healthcare methods. Firstly, TA provide unprecedented levels of convenience and accessibility, offering unparalleled convenience to patients. This innovation eliminates geographical distances and time constraints, allowing users to access healthcare services effortlessly without being constrained by geographical location or time limitations (35, 38, 62). This is highly attractive to the public as it eliminates many of the inconveniences typically associated with traditional healthcare approaches. Additionally, TA have the potential to reduce healthcare costs. For instance, by reducing travel costs and time wastage, patients can access healthcare services more economically and efficiently (6, 35, 38, 63). This is particularly significant for users who face long-distance travel or require frequent medical visits due to healthcare issues. Therefore, in this study, we hypothesize that when the public perceives TA as being superior to traditional healthcare methods, offering greater convenience and cost-effectiveness, it may enhance the public’s perceived value of TA.

*H*1. Relative advantage positively influences the public’s perceived value of TA.

#### 3.2.2 Compatibility

In the context of adopting innovative technologies, compatibility is widely regarded in academia as one of the key factors influencing the public’s acceptance of these technological innovations (64, 65). Compatibility refers to the extent to which an innovation aligns with an individual’s existing practices, beliefs, and values (28). Compatibility is a critical indicator in assessing whether a new technology aligns with a user’s current experiences and expectations (66). When an innovation or technology aligns with a user’s actual needs, beliefs, and values, users are more likely to perceive the value of that innovation or technology (67–69).

In the context of TA, compatibility implies the extent to which these applications align with users’ healthcare practices, beliefs, and values. Specifically, TA offer users convenient and flexible healthcare services, enabling them to manage health issues according to their own needs and schedules (70, 71). This flexibility makes users feel that their healthcare approach is in sync with current healthcare trends of the era. Additionally, these applications align well with users’ mobile lifestyles, providing on-the-go medical consultation and treatment opportunities (72). Importantly, TA can effectively save users time and financial costs, especially for users in rural or remote areas (6, 73). Therefore, in this study, we hypothesize that when TA align with users’ healthcare practices, beliefs, and values, the public is more likely to perceive them positively.

*H*2. Compatibility positively influences the public’s perceived value of TA.

#### 3.2.3 Complexity

Complexity is considered a crucial factor in predicting the adoption of innovation or technology (53). Complexity refers to the degree to which an innovation is perceived as relatively difficult to understand and use (28). This concept contrasts with the notion of perceived ease of use in the Technology Acceptance Model, which emphasizes the ease of use of technology (27, 53, 74, 75). A significant body of literature suggests that complexity has a negative impact on the perceived value of innovation or technology. For instance, studies have shown that the complexity of self-driving cars is generally perceived as detrimental to their perceived value (53), the complexity of digital services is negatively and significantly related to consumers’ perceived value of Personal Health Records (PHRs) (76), and the complexity of global positioning system (GPS) navigation applications is negatively associated with consumers’ perceived value (77).

In the context of this study, complexity can be defined as the degree to which the public perceives remote healthcare applications as difficult to understand and use. For individuals who are less familiar with or skilled in using technology, the complexity of remote healthcare applications may pose a barrier, contributing to the digital divide in information technology adoption (16, 18, 19). Furthermore, the complexity of remote healthcare applications may lead to difficulties in their correct usage, resulting in issues related to data security and personal privacy breaches (15–17). Therefore, this study hypothesizes that when the public perceives remote healthcare applications as being difficult to understand and use, it may decrease the perceived value of these applications.

*H*3. Complexity has a negative impact on the perceived value of TA among the public.

#### 3.2.4 Trialability

Trialability refers to the degree to which an innovation can be experimented with on a limited basis before full adoption (28). Empirical studies have shown a strong association between trialability and user acceptance of innovations or technologies (53, 65, 78). Users can assess the functionality and performance of a product by trying it out or testing it, thereby increasing their confidence in the product or service and reducing uncertainty, ultimately enhancing the perceived value of the product or service (79).

In the context of this study, trialability refers to whether a TA provides an opportunity for potential users to experiment, test, or evaluate the features, functionality, and performance of the application on a limited basis before adoption. The core concept here is that by offering free trial periods or demo versions, TA enable users to gain a comprehensive understanding of the application before actual use. This, in turn, reduces user uncertainty about this innovation and helps improve the perceived value of the application (80). Therefore, this study hypothesizes that when the public perceives that a TA can be tested, evaluated, or experimented with before use, it may enhance the public’s perceived value of the TA.

*H*4. Trialability has a positive impact on the public’s perceived value of TA.

#### 3.2.5 Observability

Observability refers to the extent to which the results of using an innovation can be observed and communicated to others (28). In the process of innovation adoption, observability is a critical driving factor because people are generally more inclined to adopt new methods or technologies that bring about clear positive outcomes and observable changes (27, 65). When the positive outcomes of an innovation can be easily seen, understood, and shared, it is more likely to be adopted (57). This observability helps build trust, generate interest, and reduce adoption risks, thereby enhancing users’ perceived value of the innovation (81, 82).

In the context of this study, observability refers to the extent to which the effects of a TA are easily visible to others. TA, as software, are relatively easy to observe because they can be readily seen, downloaded, and used on smart devices (83–85). When users can intuitively perceive the positive effects of such an application, such as improvements in healthcare, convenience, time and resource savings, and better connections with healthcare professionals, they are more likely to trust and adopt the application (6, 86). This observability not only allows individuals to clearly see the benefits of the application but also reduces potential uncertainty and risks, thereby enhancing the motivation for adoption (87). Therefore, in our study, we hypothesize that when the public perceives that the effects of a TA are easily observable by others, it may increase their perceived value of the TA.

*H*5. Observability has a positive impact on the public’s perceived value of TA.

#### 3.2.6 Perceived value and public acceptance

According to the PVT, products or services with higher perceived value are more likely to be accepted by the public (43, 53). Extensive literature research has shown that perceived value is often a key influencing factor in consumer behavior (88, 89). Therefore, when the public perceives that a product or service has a higher perceived value, they are more likely to choose options with high perceived value (90, 91). At the same time, perceived value can reduce decision uncertainty because the public believes that selecting products or services with high perceived value is more likely to meet their needs (92, 93). For example, empirical research has clearly indicated that perceived temporal value positively influences the public’s acceptance of autonomous vehicles (94). Perceived value has also been confirmed as a key predictor of the public’s willingness to purchase green homes (GHs) (95) and as a predictor of the public’s willingness to invest in sponge city plans (96).

In the context of this study, public acceptance refers to whether the public is willing to adopt or use TA in the future (97). According to the theory of perceived value, perceived value will become an important indicator for evaluating the extent to which the public accepts TA. Therefore, we hypothesize that when the public perceives that TA are valuable in terms of utility value, emotional value, and social value, they are likely to accept the application.

*H*6. Perceived value has a positive impact on the public’s acceptance of TA.

## 4 Empirical research

### 4.1 Questionnaire and survey

We designed a questionnaire based on existing research and the practical context of TA. The questionnaire employed a multi-item approach, with each item measured using a 5-point Likert scale ranging from 1 for “strongly disagree” to 5 for “strongly agree.” Appendix A provides a detailed list of the measurable items for each construct. These questionnaire items were adapted from published literature and modified to fit the context of this study. To ensure the quality of the questionnaire, it underwent a review by experts in the field of information management systems, and a pre-test was conducted with 20 master’s and doctoral students from the School of Global Business at Yeungnan University.

**Figure 2:**
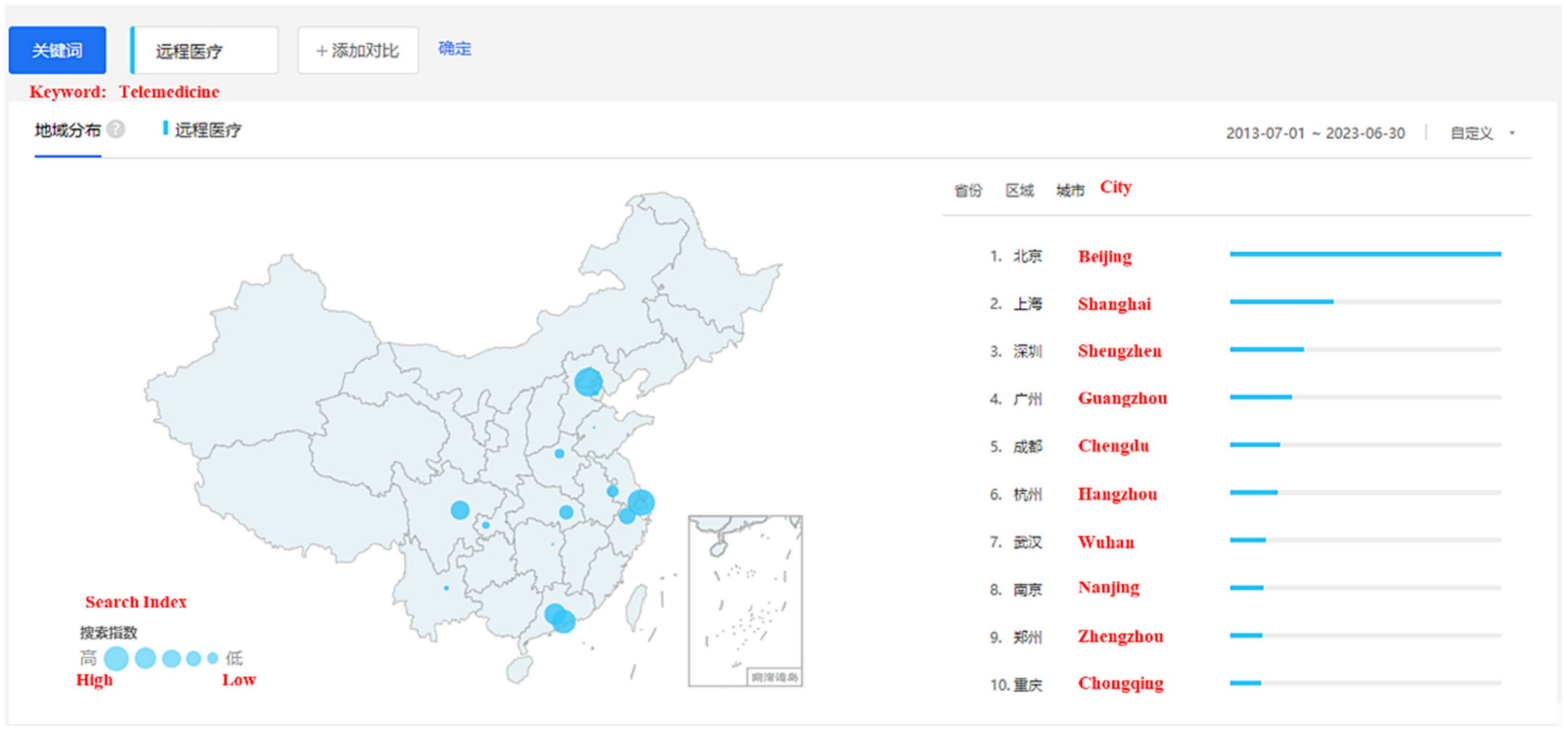
Level of interest in telemedicine across major cities in China.

China, as one of the most populous countries globally, implies a vast pool of users and potential participants for surveys on TA, thus providing a broader sample and more comprehensive insights. According to the data provided by Baidu Index from 2013 to 2023, over the past decade, residents of Beijing have shown the highest level of interest in telemedicine, as illustrated in Figure 2. Therefore, this study designates consumers residing in Beijing who have previously used TA as the targeted participants for investigation. Since the measurement items of the questionnaire were originally developed in English, we first translated the English version of the questionnaire into Chinese and then back-translated it into English to ensure readability and equivalence of meaning. Subsequently, two language experts proficient in both English and Chinese reviewed both versions to confirm the absence of any discrepancies or misunderstandings.

Prior to distributing the questionnaires, we sought the advice of members of the school’s Scientific Ethics Committee to ensure the absence of ethical issues in the survey. The survey for this study was conducted simultaneously at four transportation hubs in Beijing, namely Wangjing West, Dongzhimen, Beijing South Station, and the Beijing Zoo. The selection of survey locations was random, with each transportation hub assigned four university student assistants whose task was to intercept passengers on the streets and inquire if they resided in Beijing and were willing to participate in the survey. Only if both questions were answered affirmatively were they allowed to complete the questionnaire either in paper format or by scanning a QR code online. As a token of appreciation for participating in the survey, we provided a small gift to each participant. The survey was conducted from July 10, 2023, to July 14, 2023. In total, we collected 523 survey questionnaires, and after eliminating ineligible and invalid questionnaires, we used 387 valid questionnaires for further analysis.

### 4.2 Demographic characteristics of respondents

Table 1 presents the demographic profile of the 387 respondents. The proportion of female respondents (*N* = 265, 68.5%) was higher than that of male respondents (*N* = 122, 31.5%). The age of the respondents was predominantly between 18 and 39 years (*N* = 302, 78%). Among all respondents, 333 individuals (86%) had received higher education. The monthly income of respondents was primarily between 5,000 RMB and 15,000 RMB (*N* = 290, 75%).

**Table 1:** Demographic characteristics of respondents.

Respondent characteristics	*N* = 387	%
Gender		
Female	265	68.5%
Male	122	31.5%
Age (in years)		
18–29	163	42
30–39	139	36
40–49	58	15
50 years or above	27	7
Education		
High School or Below	54	14
College Degree	182	47
Master’s or Doctorate Degree	151	39
Income (Per month)		
Less than 5,000 RMB	28	7.2
5,001 to 10,000 RMB	154	39.8
10,001 to 15,000 RMB	136	35.2
More than 15,000 RMB	69	17.8

Based on the demographic data of the respondents, it is noteworthy that many respondents are female. However, we believe that this gender distribution is unlikely to significantly impact the quality of further data analysis. Firstly, previous research has concluded that females tend to be more actively engaged in questionnaire surveys compared to males (98, 99). Secondly, through an analysis of the characteristics of individuals who show interest in telemedicine on Baidu Index (2023 baseline), we found that many of them are female, accounting for 52.87% of the total. Therefore, gender difference is not a central focus of this study, and we believe that the respondent group is representative in this regard.

In addition, the age of participants in our study is primarily distributed between 18 and 39 years old. Through an analysis of the age distribution of individuals interested in telemedicine on Baidu Index (2023 baseline), we found that the highest proportions are in the age groups of 20–29 years (38.61%) and 30–39 years (24.75%), totaling 63.36%. This aligns closely with our survey results (78%). Therefore, we believe that the age distribution is unlikely to significantly impact the quality of further data analysis.

Furthermore, our study reveals that 86% of the participants have received higher education. According to the results of the seventh national population census by the National Bureau of Statistics of China,^1^ the population born between 1980 and 2004 has a higher education rate of 65.55%. Meanwhile, the proportion of the permanent residents in Beijing with higher education is 42%. Taking these data into consideration, we conclude that the distribution of educational backgrounds is unlikely to have a significant impact on the quality of further data analysis.

### 4.3 Analytical method

This study first conducted demographic analysis using SPSS and subsequently employed VB-SEM (Variance-Based Structural Equation Modeling) along with the corresponding application of PLS-SEM 4.0 to validate the hypotheses proposed in this research.

### 4.4 Bias test

To mitigate non-response bias in demographic data, a paired t-test was employed to examine data from 20 individuals who completed the questionnaire both at the earliest and latest stages. The validation results indicated no significant differences.

Common method bias is a common concern in questionnaire surveys. Common method bias in PLS-SEM was measured based on FLL-VIF (100, 101), with all VIF values being below 3.3. The results of testing methods suggest that Common method bias is not a significant issue in this study.

### 4.5 Measurement model test

We assessed the measurement model through various criteria, including composite reliability (CR), average variance extracted (AVE), discriminant validity, and outer loadings.

As shown in Table 2, the composite reliability for each variable exceeded 0.7, and Cronbach’s alpha was also above 0.7, indicating satisfactory internal consistency of the data in this study. Additionally, the AVE values for all variables exceeded 0.5, and the outer loadings were above 0.7, confirming the convergent validity of the data (102).

**Table 2:** Reliability and validity coefficients for constructs.

Latent variable	Item	Loading	Mean (SD)	Cronbach’s a	CR	AVE
RA	RA 1	0.876	3.210(1.049)	0.851	0.910	0.770
	RA 2	0.891				
	RA 3	0.866				
CB	CB 1	0.879	2.977(1.104)	0.856	0.912	0.775
	CB 2	0.918				
	CB 3	0.843				
CX	CX 1	0.885	2.952(1.064)	0.889	0.923	0.750
	CX 2	0.876				
	CX 3	0.855				
	CX 4	0.848				
TR	TR 1	0.890	2.955(1.087)	0.864	0.916	0.785
	TR 2	0.877				
	TR 3	0.891				
OB	OB 1	0.886	3.037(1.061)	0.886	0.921	0.745
	OB 2	0.854				
	OB 3	0.850				
	OB 4	0.861				
PV	PV 1	0.844	3.586(0.699)	0.801	0.863	0.613
	PV 2	0.789				
	PV 3	0.731				
	PV 4	0.764				
PA	PA 1	0.905	3.775(0.666)	0.717	0.821	0.607
	PA 2	0.705				
	PA 3	0.711				

RA, Relative Advantage; CB, Compatibility; CX, Complexity; TR, Trialability; OB, Observability; PV, Perceived value; PA, Public Acceptance.

Table 3 presents the results of Fornell and Larcker’s Test and the Heterotrait-Monotrait ratio (HTMT) Test, which were used to evaluate discriminant validity. The HTMT values among variables were below the threshold of 0.85, and the square root of each variable’s AVE was greater than its correlations with other variables, as recommended by Hair et al. (102). These analyses collectively indicate that this study possesses good composite reliability, convergent validity, and discriminant validity.

**Table 3:** Discriminant validity.

Fornell-Larcker Criterion							
	CB	CX	OB	PA	PV	RA	TR
CB	**0.881**						
CX	−0.027	**0.866**					
OB	−0.016	0.043	**0.863**				
PA	0.232	0.347	0.342	**0.779**			
PV	0.177	0.319	0.303	0.498	**0.783**		
RA	−0.059	−0.031	−0.002	0.176	0.235	**0.878**	
TR	0.015	−0.018	0.024	0.218	0.194	0.009	**0.886**
Heterotrait-Monotrait Ratio							
	CB	CX	OB	PA	PV	RA	TR
CB							
CX	0.063						
OB	0.061	0.048					
PA	0.263	0.352	0.385				
PV	0.179	0.342	0.321	0.523			
RA	0.074	0.038	0.026	0.170	0.254		
TR	0.027	0.04	0.042	0.238	0.184	0.045	

RA, Relative Advantage; CB, Compatibility; CX, Complexity; TR, Trialability; OB, Observability; PV, Perceived value; PA, Public Acceptance.

Model fit in this study was assessed using the SRMR (Standardized Root Mean Square Residual) value. In PLS-SEM, an SRMR value below 0.08 is considered indicative of acceptable model fit (103). In this study, the SRMR value for the model was found to be 0.064, indicating that the model exhibits acceptable fit.

Furthermore, we assessed the issue of multicollinearity, and all variables had VIFs (Variance Inflation Factors) below 3.3. Therefore, multicollinearity is not a major concern in this study.

### 4.6 Structural model test

When validating the inner model, variables related to the explained variance (R^2^), size effect (f^2^), and predictive relevance (Q^2^) were investigated (5). Through an analysis of key parameters, including Path Coefficient (*β* > at 0.200), t-value > at 1.96, and value of *p* < at 0.05 (see Table 4), the relationships among these variables were examined. For Public Acceptance, the R2 value is 0.255, indicating that the research model can account for a portion of the variance in the intention to use. Specifically, Perceived value significantly and positively influences Public Acceptance (*β* = 0.498, *t* = 15.142, *p* = 0.000), as shown in Figure 1. This result supports Hypothesis H6. However, the impact of Gender, Age, Education, and Income on the public’s acceptance of TA is neither direct nor significant, thus failing to support the hypothesis that controlling for these variables influences public acceptance.

**Table 4:** Test of hypotheses (O=Supported, X = Not Supported).

Variable	Predictor construct	*R^2^*	f^2^	β	STDEV	T Statistics	*P* values	Q^2^	Result
Perceived value		0.323						0.161	
	Relative Advantage		0.096	0.256	0.040	6.395	0.000		O
	Compatibility		0.060	0.202	0.042	4.841	0.000		O
	Complexity		0.154	−0.323	0.039	8.287	0.000		O
	Trialability		0.052	0.188	0.046	4.060	0.000		O
	Observability		0.122	0.288	0.040	7.176	0.000		O
Public acceptance		0.255						0.124	
	Perceived value		0.326	0.498	0.033	15.142	0.000		O
	Gender		0.004	−0.130	0.102	1.274	0.203		X
	Age		0.005	−0.126	0.104	1.210	0.226		X
	Education		0.005	−0.105	0.078	1.346	0.176		X
	Income		0.001	0.208	0.109	1.911	0.056		X

Further analysis reveals that the model explains 32.3% of the perceived value determined by five Predictor construct. Confirmation of the size effect (f^2^) of these five Predictor construct a clear hierarchy in their impacts, with Complexity, Observability, Relative Advantage, Compatibility, and Trialability ranking from highest to lowest. Consequently, Observability, Relative Advantage, Compatibility, and Trialability were found to have significant positive effects on Perceived value, while Complexity exhibited a significantly negative impact, confirming support for Hypotheses H1, H2, H3, H4, and H5.

The blindfolding procedure of SmartPLS4 was employed to test the predictive relevance of the model (Q^2^), with all values exceeding 0, thus confirming the predictive relevance of Perceived value (0.161) and Public Acceptance (0.124). Ultimately, as shown in Table 4, all hypotheses in this study received support, while the influence of control variables (Gender, Age, Education, Income) on the public acceptance of TA was not supported.

### 4.7 Mediation analysis

Given the non-normal distribution of the data in this study, we employed the mediation analysis method proposed by Hair Jr. et al. (104) and Bollen (105) to verify the mediating effects. They suggest that in PLS-SEM, validating mediating effects can be based on the following three steps:

Firstly, it is necessary to confirm that the direct effects between the independent variable (IV) and the dependent variable (DV) are significant. If there are no effective direct effects, the influence of the mediating variable may not hold (105). The direct effects between the independent variables and the dependent variable in this study are shown in Table 5. Relative Advantage (*β* = 0.227, *p* < 0.001), Compatibility (*β* = 0.229, *p* < 0.001), Complexity (*β* = −0.360, *p* < 0.001), Trialability (*β* = 0.227, *p* < 0.001), and Observability (*β* = 0.338, *p* < 0.001) all exhibited significance on Public Acceptance.

**Table 5:** Significance analysis of direct effects of independent variables.

Hypothesis	β	STDEV	T Statistics	*P* values
RA→PA	0.227	0.057	3.943	0.000
CB→PA	0.229	0.044	5.257	0.000
CX→PA	−0.360	0.037	9.729	0.000
TR→PA	0.227	0.044	5.130	0.000
OB→PA	0.338	0.038	8.968	0.000

RA, Relative Advantage; CB, Compatibility; CX, Complexity; TR, Trialability; OB, Observability; PV, Perceived value; PA, Public Acceptance.

Furthermore, the indirect effects between the independent variable (IV), mediator (Mediator), and dependent variable (DV) must exhibit statistical significance (105). Building upon the significant results observed in the first step, where Relative Advantage, Compatibility, Complexity, Trialability, and Observability exhibited significance on Public Acceptance, we verified the indirect effects between the independent variables (Relative Advantage, Compatibility, Complexity, Trialability, Observability), the mediator (Perceived value), and the dependent variable (Public Acceptance). The results are presented in Table 6. Relative Advantage (*β* = 0.239, *p* < 0.001), Compatibility (*β* = 0.194, *p* < 0.001), Complexity (*β* = −0.323, *p* < 0.001), Trialability (*β* = 0.259, *p* < 0.001), and Observability (*β* = 0.307, *p* < 0.001) all exhibited statistical significance on Perceived value.

**Table 6:** Significance of indirect effects of the mediator.

Hypothesis	β	STDEV	T Statistics	P values
RA→PV	0.239	0.043	5.540	0.000
CB→PV	0.194	0.044	4.387	0.000
CX→PV	−0.323	0.041	7.822	0.000
TR→PV	0.259	0.054	4.762	0.000
OB→PV	0.307	0.042	7.370	0.000
PV→PA	0.488	0.033	14.729	0.000

RA, Relative Advantage; CB, Compatibility; CX, Complexity; TR, Trialability; OB, Observability; PV, Perceived value; PA, Public Acceptance.

The indirect effects, total indirect effects, and significance results are presented in Table 7. Perceived value has a significant mediating effect on Relative Advantage (*β* = 0.117, *p* < 0.001), Compatibility (*β* = 0.095, *p* < 0.001), Complexity (*β* = −0.159, *p* < 0.001), Trialability (*β* = 0.126, *p* < 0.001), and Observability (*β* = 0.150, *p* < 0.001) in relation to public acceptance.

**Table 7:** Significance testing of indirect effects.

Hypothesis	Indirect effect 1	Indirect effect 2	Indirect total effect	STDEV	T statistics
A→B→C	A→B	B→C			
RA→PV→PA	0.239	0.488	0.117	0.027	4.333^***^
CB→PV→PA	0.194	0.488	0.095	0.026	3.654^***^
CX→PV→PA	−0.323	0.488	−0.158	0.026	−6.077^***^
TR→PV→PA	0.259	0.488	0.126	0.029	4.345^***^
OB→PV→PA	0.307	0.488	0.150	0.027	5.556^***^

*p < 0.05, **p < 0.01, ***p < 0.001, RA, Relative Advantage; CB, Compatibility; CX, Complexity; TR, Trialability; OB, Observability; PV, Perceived value; PA, Public Acceptance.

Finally, the Variance Accounted For (VAF) values were calculated to determine the magnitude of the mediating effects. If the VAF is below 20%, it is considered as no mediation; if it falls between 20 and 80%, it is considered as partial mediation; and if it exceeds 80%, it is regarded as complete mediation (104). The validation results of the VAF values are presented in Table 8, indicating that Perceived value exhibits partial mediation for Relative Advantage (VAF = 0.340, 20% ≤ 34% ≤ 80%), Compatibility (VAF = 0.293, 20% ≤ 29.3% ≤ 80%), Complexity (VAF = 0.305, 20% ≤ 30.5% ≤ 80%), Trialability (VAF = 0.357, 20% ≤ 35.7% ≤ 80%), and Observability (VAF = 0.307, 20% ≤ 30.7% ≤ 80%) in relation to public acceptance.

**Table 8:** Media effect size test results.

Hypothesis	Indirect total effect	Direct effect	Total effect	VAF	Media strength test		
					Completely (VAF > 80%)	Partial (20% ≤ VAF ≤ 80%)	None (VAF < 20%)
RA→PV→PA	0.117	0.227	0.344	0.340		O	
CB→PV→PA	0.095	0.229	0.324	0.293		O	
CX→PV→PA	−0.158	−0.360	−0.518	0.305		O	
TR→PV→PA	0.126	0.227	0.353	0.357		O	
OB→PV→PA	0.150	0.338	0.488	0.307		O	

RA, Relative Advantage; CB, Compatibility; CX, Complexity; TR, Trialability; OB, Observability; PV, Perceived value; PA, Public Acceptance.

## 5 Discussion and conclusion

### 5.1 Key findings

TA play an increasingly vital role in the current healthcare landscape. With the rapid advancement of digital technology, these apps offer patients convenient, efficient, and cost-effective medical services. However, despite their significant potential, there has been a lack of comprehensive research addressing how the public’s acceptance of TA can be theoretically explained. Therefore, this study aimed to delve into the motivations and mechanisms influencing the public’s acceptance of TA from the perspectives of innovation diffusion theory and PVT.

Firstly, the empirical study demonstrates that all five elements of innovation diffusion significantly influence the public’s perceived value of TA. This finding aligns with the argument in this study that the evaluation of innovation characteristics by the public affects their perceived value, including utilitarian value, emotional value, and social value (30–32). Specifically, relative advantage has a significantly positive impact on the public’s perceived value of TA, validating that the relative advantage of a product or service can significantly influence consumers’ perceptions of its value (59–61). This implies that the actual advantages of TA over traditional healthcare methods in terms of efficiency, cost, time, and other aspects significantly influence the public’s perceived value (6, 35, 38, 63).

Compatibility also has a positive impact on the public’s perceived value of TA, further emphasizing that when innovation or technology aligns with users’ actual needs, beliefs, and values, they are more likely to perceive its value (67–69). This implies that TA are aligned with users’ healthcare practices, beliefs, and values.

However, complexity has a negative impact on the public’s perceived value of TA. This result aligns with previous research indicating that the complexity of innovations such as self-driving cars can adversely affect their perceived value (53). Additionally, studies have shown a negative relationship between the complexity of digital services and consumers’ perceived value of personal health records (PHRs) (76), as well as the complexity of Global Positioning System (GPS) navigation apps and consumer perceived value (77). This can be explained by the notion that the complexity of TA can lead to confusion among the public, thereby reducing their perceived value.

Furthermore, trialability has a positive impact on the public’s perceived value of TA. This result is consistent with the determinants of the trialability of mHealth apps, which involve assessing whether mobile health applications meet users’ adoption standards (27). Additionally, it aligns with the research findings on the trialability of mHealth apps among the Y generation in Bangladesh, where it is considered a crucial antecedent to the intention of mobile health application usage (84). It suggests that when the public can assess the effectiveness and benefits of a medical app through trial or firsthand experience, they are more likely to be interested in it, thus perceiving its value.

Observability also has a positive impact on the public’s perceived value of TA. This result reinforces the notion that people are generally more inclined to adopt new methods or technologies that bring about noticeable positive outcomes and observable changes (27, 65). This observability helps build trust, spark interest, and reduce adoption risks, thereby enhancing users’ perceived value of innovations (81, 82). A reasonable explanation is that when users can intuitively see how a medical app improves their health or quality of life, they are more likely to perceive its value.

Secondly, this study employed the theory of perceived value as a theoretical framework and empirically analyzed the positive impact of perceived value on the public’s acceptance of TA. It further delved into the mediating effects of the five elements (namely relative advantage, compatibility, complexity, trialability, and observability) during the innovation diffusion process on the public’s acceptance of TA. The research findings indicate that perceived value has a positive impact on the public’s acceptance of TA. This finding aligns with the theory of perceived value, which posits that if TA can offer users the highest utility, it will enhance the public’s acceptance of these apps (29). Furthermore, this result is consistent with previous research, which has demonstrated that perceived value is a crucial antecedent influencing the public’s acceptance of various innovations in different domains, including private autonomous vehicles (94), green housing (GHs) (95), and sponge city planning (SCP) (96).

Furthermore, perceived value plays a partial mediating role in explaining the influence of the five elements (i.e., relative advantage, compatibility, complexity, trialability, and observability) in the innovation diffusion process on the public’s acceptance of TA. Specifically, perceived value mediates the relationship between these factors and the public’s acceptance of TA by shaping their cognitive perceptions of relative advantage, compatibility, complexity, trialability, and observability. Whether the public perceives TA as valuable to them will to some extent determine their willingness to adopt this innovation.

Finally, it is important to note that this study did not find sufficient evidence to support the impact of control variables (gender, age, education, income) on the public acceptance of TA. This conclusion contrasts with the meta-analysis conducted by Calegari et al. (106), which indicated significant effects of gender and age on the intention to use mobile health technologies (106). One possible explanation is that the participants in this study were predominantly female, and the age distribution was concentrated primarily between 18 and 39 years old. Therefore, limited variations in demographic characteristics may have resulted in an inability to observe the influence of control variables on the acceptance of TA.

### 5.2 Theoretical contributions

This study provides significant contributions to the academic field. Firstly, we successfully bridge the current theoretical gap in explaining the public’s acceptance of TA by introducing and integrating two theories: the Innovation Diffusion Theory and the PVT. This in-depth understanding of the factors influencing the public’s acceptance of TA offers a solid theoretical foundation for the promotion of TA.

Secondly, by delving into the impact of the five key elements of the Innovation Diffusion Theory (relative advantage, compatibility, complexity, trialability, and observability) on the public’s perceived value of TA, we unveil the crucial mechanisms behind audience acceptance behavior. This analysis helps us better understand why some individuals are more willing to accept TA while others may exhibit skepticism or resistance.

Thirdly, we explore the influence of perceived value on the public’s acceptance of TA, particularly in terms of the theoretical explanations for the public’s perceived value of these apps. Despite the significant relevance of TA in the field of healthcare innovation, a comprehensive understanding of their perceived value remains underdiscussed. By delving into this aspect, our study provides valuable insights for future research and practice.

Finally, we analyze the mediating role of perceived value in the mechanism between the five elements of innovation diffusion and public acceptance. This contributes to a clearer understanding of the role of perceived value in the decision-making process and why certain elements may be crucial for the public’s acceptance of TA through their influence on perceived value. This mediation analysis offers profound insights into explaining the critical importance of some elements for the public’s acceptance of TA.

### 5.3 Practical contributions

Furthermore, this study provides significant practical contributions. Firstly, by offering robust empirical support, this research lays a crucial foundation for the current landscape of digital healthcare innovation. Our findings not only enhance the acceptance of TA but also offer valuable practical insights for the design and promotion of other digital healthcare tools. This contributes to advancing the digital transformation in the healthcare sector, improving the quality, accessibility, and efficiency of healthcare services.

Secondly, this study offers powerful strategic guidance for developers of TA. We provide specific recommendations based on the DOI theory and the PVT to improve product designs, ensuring that the apps align more closely with user needs. These recommendations include simplifying user interfaces, providing clear usage guidelines, and enhancing user support services. Additionally, we encourage developers to actively collaborate with healthcare institutions to ensure that their apps align with the needs and processes of the healthcare system, thereby increasing the likelihood of adoption.

Thirdly, this study provides valuable insights for healthcare professionals, including doctors, nurses, and other healthcare practitioners. It helps them better understand patient perspectives and needs regarding TA, thus improving interactions with patients. By effectively communicating the benefits of the apps, healthcare professionals can increase patient acceptance of new technologies, strengthen communication and trust between patients and healthcare practitioners, and enhance treatment compliance and outcomes.

Lastly, widespread adoption of TA can lead to cost savings for the overall healthcare system. These apps reduce the need for outpatient and inpatient treatments, thus conserving medical resources and reducing costs. This cost-reduction effect is critical for healthcare institutions to provide more cost-effective medical services and alleviate the financial burden on patients, contributing to the sustainability of the healthcare system and the economic accessibility of patients. Additionally, it is worth noting that promoting the widespread use of TA also contributes to bridging the digital divide. This includes improving digital health education, internet connectivity, and providing affordable digital devices to ensure that diverse social groups can fully utilize TA, thereby increasing the accessibility and equity of healthcare services and promoting overall societal health and public health.

### 5.4 Limitations and future research

While this study has yielded important findings and practical contributions, it also has certain limitations that provide valuable directions for future research.

Firstly, the sample in this study primarily consisted of residents from Beijing, China. Consequently, the research results may be influenced by regional and cultural factors. Future research could expand the sample scope to include participants from a wider range of regions and diverse cultural backgrounds, especially samples from rural or remote areas, to enhance the external validity of the study.

Secondly, this study employed a cross-sectional survey design, limiting the in-depth understanding of the TA acceptance process. Future research could employ longitudinal study designs to track participants over time, providing a more comprehensive understanding of their acceptance processes, including long-term usage and sustained acceptance.

Furthermore, the participants in this study are predominantly aged between 18 and 39, leading to an insufficient sample representation that cannot fully capture the diversity of the entire population, especially in terms of the older adult demographic. The significant limitation arises from the lack of participants in specific age groups, preventing a comprehensive understanding of the specific needs and trends of the older adult population in the area under investigation. Additionally, the absence of individuals from specific age groups may overlook the potential impact of the digital divide on the study results. For instance, older individuals might face unequal access to information and communication due to limitations in technology use, introducing biases to the research findings. Therefore, for a more comprehensive understanding of the experiences and needs of individuals across different age groups and digital literacy levels in the relevant field, future research should strive to enhance sample representation to ensure broader applicability of research outcomes.

Finally, this study primarily relied on the DOI theory and PVT, which have provided strong support for explaining acceptance behavior. However, future research could consider integrating other theories such as the Unified Theory of Acceptance and Use of Technology or Social Cognitive Theory for a more comprehensive understanding. Additionally, further exploration of other potential factors, such as security and privacy concerns, and their impact on TA acceptance could be investigated.

## Data availability statement

The raw data supporting the conclusions of this article will be made available by the authors, without undue reservation.

## Author contributions

DL: Conceptualization, Data curation, Formal analysis, Investigation, Methodology, Software, Validation, Visualization, Writing – original draft, Writing – review & editing. SS: Conceptualization, Investigation, Supervision, Validation, Writing – review & editing. JC: Formal analysis, Investigation, Methodology, Project administration, Supervision, Validation, Writing – review & editing.

## Footnotes

^1^http://www.stats.gov.cn/zt_18555/zdtjgz/zgrkpc/dqcrkpc/

## Appendix A

**Table:**

Factors	Serial num.	Item	Reference
Relative Advantage (RA)	RA1	Using TA is time-saving for me.	Mehra et al. (57)
	RA2	Using TA is highly efficient for me.	
	RA3	Compared to traditional healthcare methods, TA are very useful for me.	
Compatibility (CB)	CB1	I believe using TA aligns with my preferred approach.	Mehra et al. (57)
	CB2	Using TA aligns with my style.	
	CB3	Using TA aligns with my preferred way of doing things.	
Complexity (CX)	CX1	Learning to use TA is challenging for me.	Mehra et al. (57)
	CX2	It’s not easy to get TA to do what I want them to do.	
	CX3	My interactions with TA are not clear and straightforward.	
	CX4	Using TA is not convenient for me.	
Trialability (TR)	TR1	I had the opportunity to try out TA before deciding whether to adopt them.	Mehra et al. (57)
	TR2	I was able to try out TA correctly before deciding whether to adopt them.	
	TR3	I was allowed to try out TA for enough time to see what they can do.	
Observability (OB)	OB1	Others seem quite interested when they see me using TA.	Mehra et al. (57)
	OB2	People can tell that I know more about TA since I started using them.	
	OB3	Other people who use TA enjoy using them.	
	OB4	I can effortlessly explain to my friends ‘what TA are all about.’	
Perceived value (PV)	PV1	I feel that using TA can meet my healthcare needs.	Yu et al. (43)
	PV2	I feel that using TA can save costs.	
	PV3	I feel that the experience of using TA is enjoyable.	
	PV4	I feel that using TA can enhance my social image.	
Public Acceptance (PA)	PA1	Assuming that I was given the chance to use TA, I intend to use TA.	Kamal et al. (97)
	PA2	Whenever I require remote healthcare services from professionals, I am willing to use TA.	
	PA3	I plan to introduce TA to my relatives and friends.	
